# Epilepsy an Update on Disease Mechanisms: The Potential Role of MicroRNAs

**DOI:** 10.3389/fneur.2018.00176

**Published:** 2018-03-19

**Authors:** Michele Simonato

**Affiliations:** ^1^Department of Medical Sciences, University of Ferrara, Ferrara, Italy; ^2^School of Medicine, University Vita-Salute San Raffaele, Milan, Italy

**Keywords:** epilepsy, hippocampus, microRNA, gene expression, meta-analysis

## Abstract

So far, research on epilepsy mechanisms has been designed mainly using animal models and tracking down molecular mechanisms underlying seizures in that model. While this approach is clearly valuable, it can be questioned if it is the best possible. One attractive alternative approach may stem from the consideration of epilepsy as a complex disease of a very complex organ, the brain. This short review summarizes data from analyses of the alterations in expression of microRNAs and their target messenger RNAs in a specific brain subregion, the dentate gyrus of the hippocampus, in three experimental models of lesional epilepsy. The findings are discussed within the conceptual framework of complex systems.

## Introduction: Definition and Classification of the Epilepsies

The term “epilepsy” refers to a collection of diseases of different etiologies, characterized by the spontaneous and unpredictable occurrence of seizures, i.e., of transient “signs and/or symptoms due to abnormal excessive and synchronous neuronal activity in the brain” (1). In practical terms, epilepsy is identified “by any of the following conditions: (1) at least two unprovoked (or reflex) seizures occurring more than 24 h apart; (2) one unprovoked (or reflex) seizure and a probability of further seizures similar to the general recurrence risk (60% or more) after two unprovoked seizures, occurring over the next 10 years; (3) diagnosis of an epilepsy syndrome” (1).

The classification of the seizures and of the epilepsies has been recently revised by the International League against Epilepsy (2, 3). According to this new classification, seizures are subdivided in focal onset, “when originating within networks limited to one hemisphere”; generalized onset, when “originating at some point within, and rapidly engaging, bilaterally distributed networks”; and unknown onset (2). Focal onset seizures can be further classified based on awareness (focal aware seizures and focal seizures with impaired awareness); based on the motor component (motor and non-motor seizures); based on clinical evolution (focal to bilateral tonic–clonic seizures). Generalized onset seizures can be further classified based on the motor component: motor (tonic–clonic and other types of motor seizures) and non-motor (for example absence seizures).

The epilepsies are classified on the basis of the onset of seizures in focal, generalized, combined focal and generalized, and unknown. Their etiology can be genetic, structural, metabolic, infectious, immune, or again unknown (3).

## Pathogenetic Mechanisms

Experimental and clinical studies in genetic and lesional epilepsies suggest the existence of several common pathogenetic mechanisms, which include alterations in the intrinsic properties of the neuron and unbalance between excitatory (glutamate) and inhibitory (γ-aminobutyric acid, GABA) signals (4, 5). Regarding the former, genetic mutations or acquired functional alterations in Na^+^ and K^+^ channels have been identified in relevant animal models and patients with different forms of epilepsy. Regarding the latter, genetic mutations or acquired functional alterations leading to loss of function of GABAergic neurotransmission (alterations in GABA synthesis, release, or receptors) or gain of function of glutamatergic neurotransmission (alterations in glutamate receptors or reuptake) have been identified.

These findings are coherent with the known mechanisms of action of the currently available anti-epileptic drugs (AEDs (5)). In fact, many AEDs target ion channels and inhibit neuronal excitability. These targets include voltage-gated Na^+^ channels (for phenytoin, carbamazepine, valproic acid, felbamate, rufinamide, lamotrigine, lacosamide, topiramate, zonisamide, and oxcarbazepine), K^+^ channels (for retigabine) and T-type Ca^2+^ channels (for ethosuximide and valproic acid). Other AEDs target molecules at the excitatory synapse and reduce their efficiency. These targets and drugs include the synaptic vesicle glycoprotein 2A (for levetiracetam), the α2δ subunit of the voltage-gated Ca^2+^ channel (for gabapentin and pregabalin), α-amino-3-hydroxy-5-methyl-4-isoxazole propionic acid, and N-methyl-d-aspartate receptors (respectively, for perampanel and felbamate). Finally, some AEDs target inhibitory synapses and enhance their efficiency. These include tiagabine, which inhibits the GABA transporter GAT1, leading to reduced GABA uptake into presynaptic terminals and astrocytes and vigabatrin, which irreversibly inhibits the catabolic enzyme GABA transaminase, leading to accumulation of GABA in presynaptic terminals and astrocytes. The benzodiazepines and the barbiturates enhance inhibitory neurotransmission by allosterically modulating GABA_A_ receptor-mediated Cl^−^ currents. Incidentally (and interestingly), many AEDs (for example, valproic acid, lamotrigine, phenobarbital, gabapentin, felbamate, and topiramate) can act through multiple complementary mechanisms.

## Unmet Therapeutic Needs in Epilepsy

In spite of all the abovementioned AEDs, many therapeutic needs remain unmet in epilepsy (6, 7). First, current drug treatments must be taken daily even in cases when seizures are rare, and patients may even become paradoxically more exposed to the side effects of their drugs than to the signs and symptoms of their epilepsy. We need more tolerable treatments that do not impact quality of life. Second, the available AEDs can prevent seizures in about two thirds of the patients and are ineffective in some forms of epilepsy. Therefore, we need new treatments for therapy resistance patients. In addition, the available AEDs are actually purely symptomatic agents (8) that, at best, prevent or reduce seizures but do not attenuate some comorbidities (such as depression, cognitive slowing, and memory impairment), which can have a dramatic impact on the quality of life of people with epilepsy (9). Finally, another urgent therapeutic need is the identification of currently unavailable antiepileptogenic and disease modifying therapies. As stated, AEDs are actually symptomatic agents for seizures, but cannot prevent the development of epilepsy in at-risk individuals who experienced a head trauma, an episode of status epilepticus (SE), a stroke, or other epileptogenic insults.

## Identification of Disease Mechanisms

In summary, we do need to move beyond current knowledge and concepts in order to identify disease mechanisms that represent relevant therapeutic targets for improving not only seizures but also epilepsy comorbidities. So far, research on epilepsy mechanisms has been designed mainly using animal models and tracking down molecular mechanisms underlying seizures in that model. While this approach is clearly valuable, it can be questioned if it is the best possible. In fact, it has been postulated that some forms of epilepsy (so-called “system epilepsies”) depend on a dysfunction of neuronal systems and not of specific, individual elements of the systems (10). Therefore, one attractive new approach to attempt understanding the mechanisms of epilepsy may stem from the consideration of epilepsy as a complex disease of a very complex organ, the brain (11).

By definition, a complex system is “any system featuring a large number of interacting components, whose aggregate activity is non-linear (i.e. cannot be derived from the summation of the activities of the components) and typically exhibits hierarchical self-organization” (12). Therefore, the brain is a quintessential complex system. In fact, it displays a hierarchical self-organization: molecular networks in each particular cell; neurons and non-neuronal cells organized in local circuits; neuronal networks involving multiple brain areas. At all hierarchical levels, the aggregate activity of the components is non-linear and emerging properties are observed that cannot be inferred from the properties of the single components.

Traditional research approaches tend to assume a linear progression from a specific molecule to cells to integrated function. In contrast, new therapeutic approaches may be developed considering the brain as a complex network and taking into account the non-linearity of its function. The feasibility and the potential of network science applied to regulation of gene expression, activity of local neural network, and global brain function begin to be appreciated (11).

In this line of reasoning, we thought to investigate the modifications of expression of microRNAs (miRNAs) in a specific neuronal population, the dentate gyrus (DG) granule cells (GCs) of the hippocampus, in the natural history of disease in experimental models of post-SE epilepsy (13).

## Why Focusing on miRNAs and on DG GCs

MicroRNAs are small size (21–25 nucleotides) endogenous non-coding RNAs. Their main function is to degrade or repress translation of specific target messenger RNAs (mRNAs), i.e., to regulate their expression at the post-transcriptional level (14, 15). miRNAs and their mRNA targets follow a “many-to-many” mode of interaction, because each miRNA can regulate many mRNAs and each mRNA can be regulated by many miRNAs (16). More than 2,000 human miRNAs have been identified thus far and more than 50% of these are expressed in the brain. miRNAs are implicated in many brain functions that are relevant for epilepsy and epileptogenesis, including cell death, neurogenesis, and synaptic plasticity (17). In fact, several studies support the notion that miRNAs play a pathogenic role in acquired epilepsies (18, 19). Thus, defining the patterns of changes in the miRNA levels in the natural history of epilepsy and their relationship with mRNA changes seems a logical approach to attempt identifying ways to modulate neuronal function in a manner that may be more closely related to the pathogenesis of disease than simply acting on phenomenological aspects such as seizures.

However, alterations in gene expression patterns are cell specific, and performing molecular analyses on large samples of tissue containing multiple cell types generates data that are difficult to interpret, because the cell composition of the same brain area may dramatically change in the course of epilepsy due to cell death, neurogenesis, astrocytosis, and other events (20). Therefore, we decided to start analyzing a specific cell population, the DG GCs. These cells have been considered for long as a “gate” inhibiting hippocampal hyperexcitation (21). Recent evidence reinforced this hypothesis: first, optogenetic GC hyperpolarization stopped spontaneous seizures, whereas GC activation exacerbated seizures; and second, acute seizures were evoked in non-epileptic animals by optogenetic activation of GCs (22). In addition, DG GCs undergo relevant functional changes with epilepsy development, like sprouting of the mossy fibers and increased excitation (20). The DG is very often involved in seizure generation in patients with temporal lobe epilepsy, one of the most common epileptic syndromes in adults, and very often surgically removed in drug resistant patients. Finally, and quite relevant from a practical point of view, DG GCs are a compact layer of almost identical cells, easy to dissect from the rest of the tissue, and very well characterized from a developmental, neurochemical, morphological, and physiological point of view.

## miRNA Profiles in Hippocampal GCs of Rats with Pilocarpine-Induced Epilepsy and Comparison with Human Epileptic Samples

We laser-microdissected the hippocampal granule cell layer (GCL) of rats killed at different time points in the development of pilocarpine SE-induced epilepsy: latency (i.e., the time period between an epileptogenic insult and the first spontaneous seizure), first spontaneous seizure, and chronic epileptic phase. By performing miRNA microarrays on these samples, we identified 63 miRNAs that were differentially expressed at one or another time point. Distinct clusters of miRNAs separated control and chronic phase rats from those sacrificed during latency or after the first spontaneous seizure. Those miRNAs that were altered in the chronic phase of the rat model were then compared with those obtained from the laser-microdissected GCL of temporal lobe epilepsy patients who underwent respective surgery, allowing the identification of many miRNAs (miR-21-5p, miR-23a-5p, miR-146a-5p, and miR-181c-5p) that were upregulated both in the human and in the rat epileptic tissue (13, 23).

## Meta-Analysis of the Available Data Sets

Taken together, these results identify miRNA expression patterns that are associated with different phases in the natural history of epilepsy in the pilocarpine model, revealing molecular pathways that may underlie the development and the maintenance of the disease. While these miRNAs represent attractive therapeutic targets, an obvious question arises: are they model- or disease-specific? The overlaps with the human findings in the chronic stage support the notion that animal data can (at least in part) predict the human situation. In addition, an overlap could also be observed between miRNAs differently expressed in this study and those identified in other studies that employed other epilepsy models (24, 25). Thus, these particular miRNAs, whose expression changes are similar in the different data sets, are particularly attractive candidates for further investigation. However, the different miRNA studies vary in many parameters, including the brain region, the animal model, the sample size, the microarray platform, and analysis techniques. Last but not least, each of these studies is underpowered to detect relatively small changes in miRNA expression.

Therefore, to pursue a rigorous answer to the question, we undertook a meta-analysis of the changes in miRNA expression reported to occur in the DG following different epileptogenic insults (26). While meta-analyses of human research data are well established, meta-analyses of preclinical data are rare, primarily because of the small sample sizes and of the high heterogeneity between studies. Indeed, our meta-analysis was the first to be performed on preclinical epilepsy research data.

Inclusion criteria were miRNA array studies in the DG of epileptic vs. control animals. Exclusion criteria were miRNA array studies in large brain areas (including the whole hippocampus). These criteria identified three data sets that used models of epilepsy induced by different epileptogenic insults: focal electrical stimulation of the lateral nucleus of the amygdala (24); focal electrical stimulation of the angular bundle, the main afferent pathway to the hippocampus from the entorhinal cortex (25); and systemic administration of the convulsant agent pilocarpine (13). All these models are characterized by recovery from the initial insult and onset of spontaneous recurrent seizures after a latency period of apparent well being. They all imply a key involvement of the DG but *via* different pathways: direct DG activation with the angular bundle stimulation, indirect with the amygdala stimulation, and widespread activation of the brain in the pilocarpine model (27). All these three studies were underpowered to detect modest fold changes (<2.0) in miRNA expression.

We found that 176 miRNAs were detectable at some time point in any of the three studies. Therefore, we meta-analyzed the expression values of these 176 miRNAs, and identified 26 that were differentially expressed in the latent period and 5 in chronic epilepsy (Table 1). Thirteen of these were not identified in the individual studies. In the attempt to begin understanding the role of these 31 miRNAs, their mRNA targets were first predicted *in silico* using the miRWalk database, yielding (as expected) a very large number of potential targets. We then filtered these predicted targets by searching for an inverse relationship with mRNAs identified in separate epileptogenesis studies (28). We found an inverse relationship between 22 (of 26) miRNAs and 112 predicated gene targets. In addition, an inverse relationship was found between 5 (of 5) miRNAs and 29 predicated gene targets for the chronic phase in the only model that could be assessed for this phase (amygdala stimulation).

**Table 1:** Differentially expressed microRNAs in the epileptogenesis and in the chronic period, as compared with controls.

Epileptogenesis	Chronic period
Upregulated	Downregulated
miR-21-5p	*miR-130a-3p*
miR-132-3p	miR-148b-3p
miR-146a-5p	*miR-324-3p*
miR-212-3p	miR-551b-3p
miR-212-5p	miR-652-3p
miR-344b-2-3p	
Downregulated	
miR-7a-5p	
*let-7b-3p*	
*let-7d-3p*	
miR-29c-5p	
miR-33-5p	
miR-92b-3p	
*miR-101a-3p*	
*miR-136-3p*	
miR-138-5p	
miR-139-5p	
*miR-150-5p*	
*miR-153-3p*	
miR-324-5p	
miR-330-3p	
*miR-335*	
*miR-345-5p*	
*miR-383-5p*	
miR-551b-3p	
*miR-667-3p*	
*miR-3573-3p*	

*MicroRNAs in italics were not identified as differentially expressed in the individual studies*.

Analysis was further refined focusing on the epileptogenesis phase. We found that anti-correlated miRNAs and mRNAs may be part of pro- or anti-epileptic mechanisms. For example, the mRNA encoding the mitogen-activated protein kinase kinase kinase 4 was predicted target and had inverse expression relative to four miRNAs (miR-92b-3p, miR-101a-3p, miR-153-3p, and miR-3575-3p), and the mRNA coding for Map3k14 was inversely correlated with two other miRNAs (miR-7a-5p and miR-138-5p). In both these cases, downregulation of miRNAs was associated with upregulation of target genes that are thought to play a pro-epileptic role (29–33). Other anti-correlated miRNAs and mRNAs may instead be part of anti-epileptic mechanisms. For example, synapsin type 2 was inversely correlated with three miRNAs (miR-101a-3p, miR-139-5p, and miR-551b-3p), and the protein tyrosine phosphatase, non-receptor type 5 was inversely correlated with two miRNAs (miR-150-5p and miR-383-5p). In these cases, downregulation of miRNAs and upregulation of the target gene may play an anti-epileptic role (34–38).

Other transcripts that were downregulated during epileptogenesis were predicted targets of miRNAs that were upregulated. For example, the downregulated 5-hydroxytryptamine receptor 5 was a predicted target of the upregulated miR-146a-5p. The downregulated GABA receptor subunit delta was a predicted target of and inversely correlated with miR-212-5p. These changes may play a pro-epileptic role (39, 40).

Finally, we generated a network of miRNAs based on their ability to target common pathways and hypothesized a connection between pairs of miRNAs if the respective mRNA targets belonged to the same, significantly enriched pathways or gene ontology terms. In line with other findings (41, 42), this functional enrichment analysis supports a role of molecular processes implicated in neuroinflammation and synaptic function in the development of epilepsy.

While these results highlight the utility of meta-analysis in increasing the value of existing preclinical data and identifying common mechanisms across different animal models, many limitations of the study should be taken into account: (1) we compared data sets that used different microarray platforms, and therefore some miRNAs may be missing in one or another; (2) miRNAs are only one of the many factors regulating gene expression, and others (histone modifications, DNA methylations, changes in transcription factors) should be taken into account; (3) this analysis focused on a specific hippocampal subarea and cell population, the DG GCs, but other brain areas and other cell populations may be at least equally important; (4) epileptogenesis and chronic epilepsy develop differently in different models, implicating that changes in miRNAs may dynamically vary at different time points; (5) anti-correlation assumes that miRNAs decrease the levels of their target mRNAs, which is the best characterized, but not the only mechanism of miRNA action (43).

This all notwithstanding, the miRNAs identified in our meta-analysis and their predicted effects on mRNAs generate hypotheses about the molecular mechanisms underlying epilepsy that will deserve further investigation. In addition, these findings may be cross-analyzed with those generated using proteomics to further corroborate the working hypotheses, in a research model in which big data obtained with different approaches are interconnected. These investigations may ultimately lead to miRNA-based therapies, which are already under preclinical investigation and may hold some advantages over traditional drug treatments in terms of precision for specific forms of epilepsy or even for specific phases of the disease. Unfortunately, the estimated costs of these therapies would be (at least for now) extremely high. Interestingly, the number and heterogeneity of the identified miRNAs suggest that therapies targeting a single miRNA may be insufficient to interfere with the epileptogenic process. This conclusion is in line with the idea of complex networks, but does not exclude the possibility of ultimately identifying sets of new potential therapeutic targets (rather than single ones).

## Conclusion

As noticed, the studies described in this short review capture only a component of a more complex network, at a low hierarchical level. Understanding of networks not only from genetic but also from local neural and global brain data will ultimately allow identification of new targets for the treatment of seizures and epilepsy comorbidities (11).

## Author Contributions

The author confirms being the sole contributor of this work and approved it for publication.

## Conflict of Interest Statement

The author declares that the research was conducted in the absence of any commercial or financial relationships that could be construed as a potential conflict of interest. The reviewer MT and handling editor declared their shared affiliation.
